# Supplementary figures and images for: CD74 promotes the formation of an immunosuppressive tumor microenvironment in triple-negative breast cancer in mice by inducing the expansion of tolerogenic dendritic cells and regulatory B cells
Source: PLoS Biol. 2024 Nov 22;22(11):e3002905. doi: 10.1371/journal.pbio.3002905 (PMC11623796; doi:10.1371/journal.pbio.3002905)

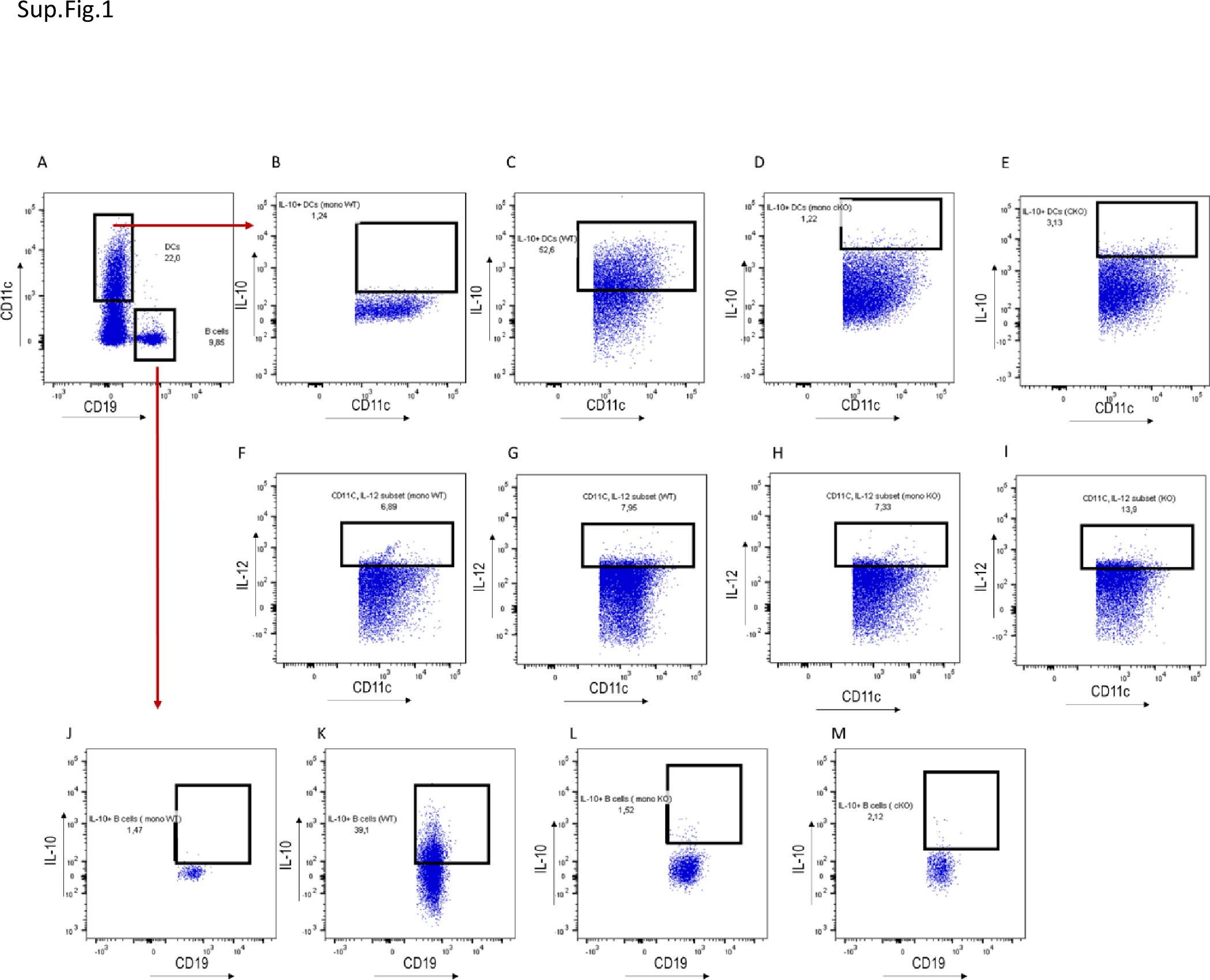

Supplement: S1 Fig — (A–M) PBMCs from the tumor site were activated with PIM and then analyzed by flow cytometry. Dead cells were excluded from analysis by Zombie Live/Dead staining. (A) DC cells were analyzed for CD11c expression after excluding LY6-C+, F4/80+, and CD19. B cells were analyzed for CD19 after excluding LY6-C+, F4/80+, and CD11c. (B–E) IL-10+ expression on DCs was measured by comparing the non-activated for either WT and CD74 -/- samples with the ones activated with PIM. (F–I) IL-12+ expression on DCs was measured by comparing the non-activated for either WT and CD74 -/- samples with the ones activated with PIM. (J–M) IL-10+ expression on B cells was measured by comparing the non-activated for either WT and CD74 -/- samples with the ones activated with PIM. The FCS files uploaded to FlowRepository (http://flowrepository.org/id/FR-FCM-Z8ES). (TIF) [file pbio.3002905.s001.tif]

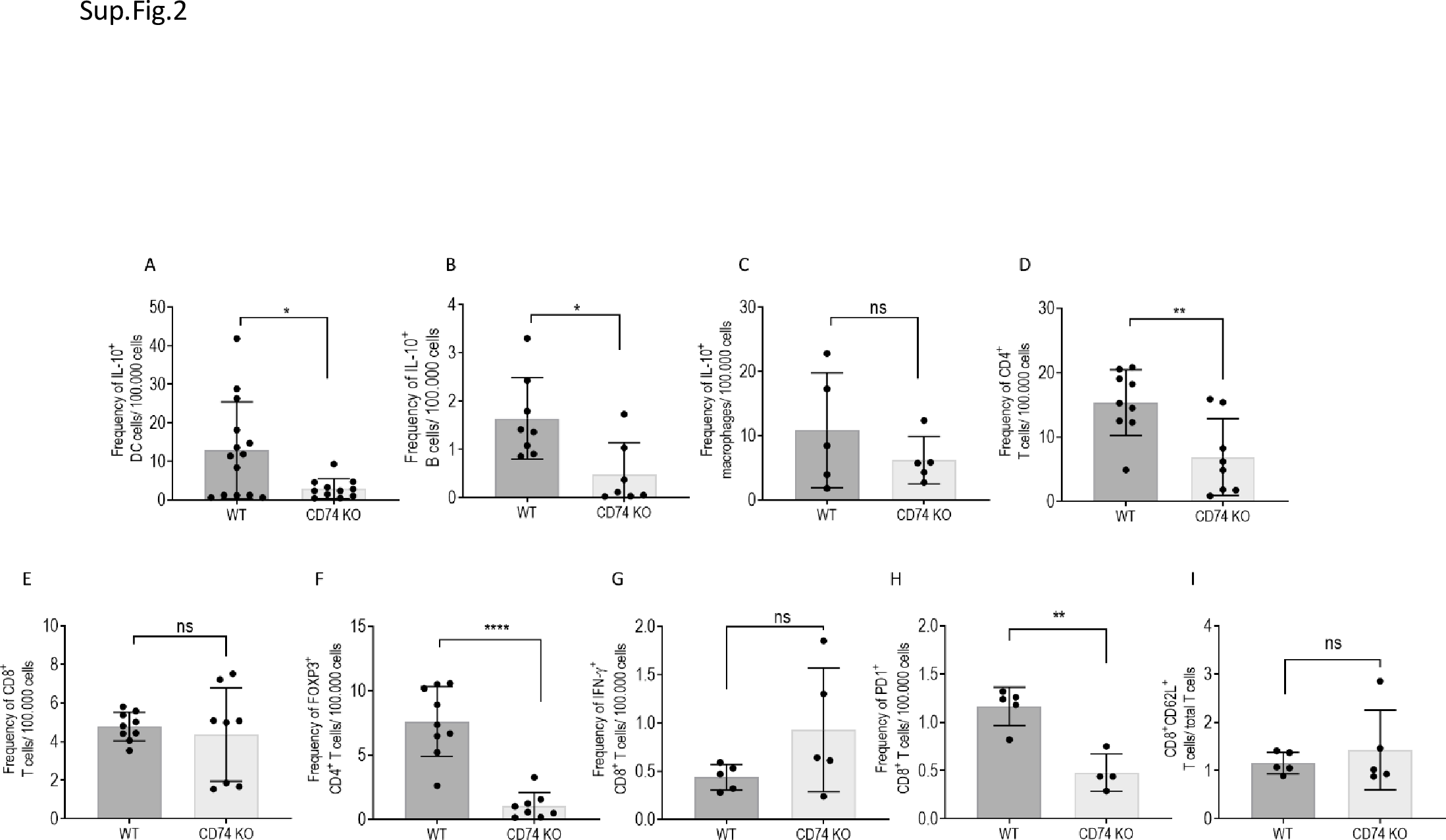

Supplement: S2 Fig — CD74 regulates the accumulation of tolerogenic immune cells in the TME. (A–I) 6 weeks old C57BL/6 and CD74-/- female mice were injected with 5 * 105 E0771 cells into each of the fourth mammary pads (total of 2 mammary pads per mouse). (A) DC cells were analyzed for CD45, CD11c, and IL-10 expression after excluding LY6-C+, F4/80+ and CD19+ cells. Graph shows the frequency of IL-10+ DCs in the tumor site (WT n = 14; CD74-/- n = 11). (B) Frequency of IL-10+ B cells out of total B cells (WT n = 8; CD74 -/- n = 7). (C) Frequency of IL-10+ macrophages after excluding monocytes and DCs (WT n = 5; CD74-/- n = 5). (D) Frequency of CD4+ T-cells (WT n = 9; CD74-/- n = 8). (E) Frequency of CD8+ T-cells (WT n = 9; CD74-/- n = 8). (F) Frequency of FOXP3+ T cells out of total CD4+ T cells (WT n = 9; CD74-/- n = 8). (G) Frequency of IFN-γ+ T cells out of total CD8+ T cells (WT n = 5; CD74-/- n = 4). (H) Frequency of PD1+ T cells out of total CD8+ T cells (WT n = 5; CD74-/- n = 4). (I) Frequency of CD62L+ T cells out of total CD8+ T cells (WT n = 5; CD74-/- n = 5). ns p > 0.05, * p < 0.05, ** p < 0.005, ****p < 0.00005. (TIF) [file pbio.3002905.s002.tif]

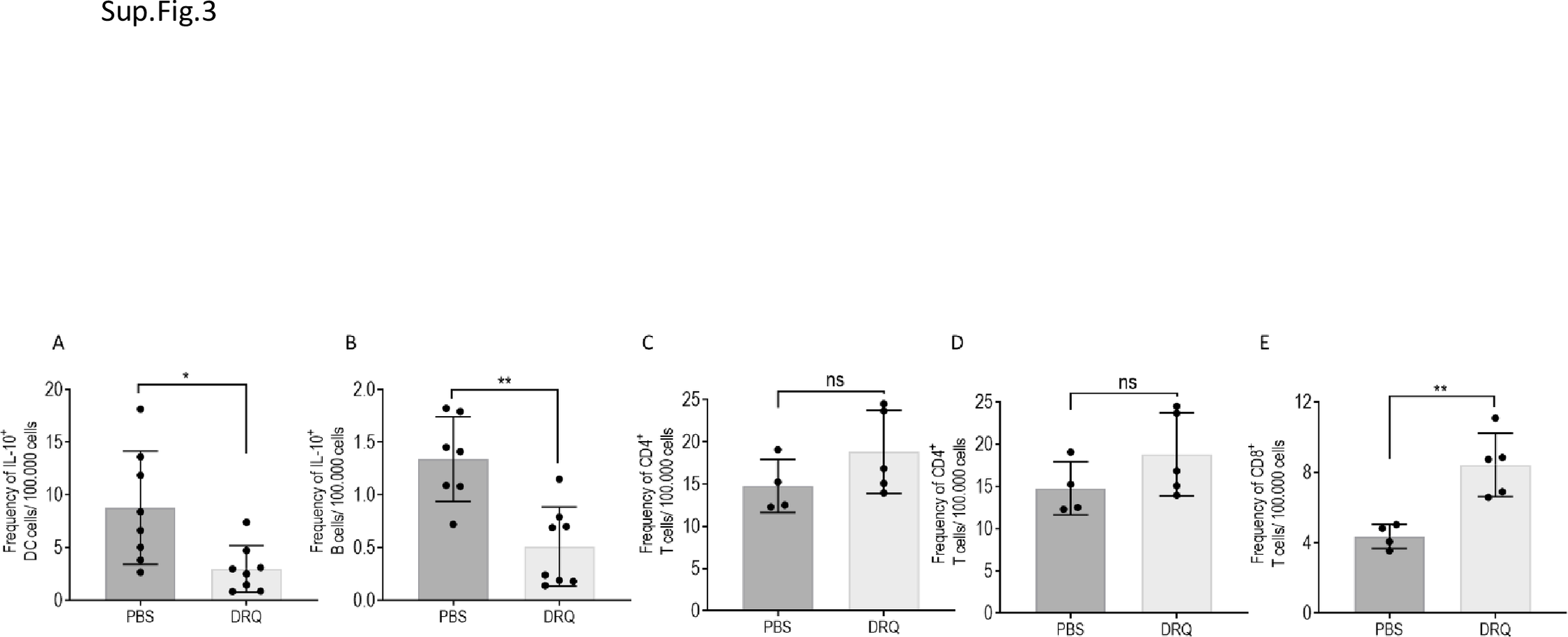

Supplement: S3 Fig — The CD74 blocker DRQ restores the immunogenicity of the TME. (A–E) Six weeks old C57BL/6 female mice were injected with 5 * 105 E0771 cells into each of the fourth mammary pads (total of 2 mammary pads per mouse). On days 10, 11, 12, 13, 14, after tumor implantation, DRQ was intravenously injected. (A) DC cells were analyzed for CD45, CD11c, and IL-10 expression after excluding LY6-C+, F4/80+ and CD19+ cells. Graph shows the frequency of IL-10+ DCs in the tumor site (PBS n = 8; DRQ n = 8). (B) Frequency of IL-10+ B cells out of total B cells (PBS n = 7; DRQ n = 8). (C) Frequency of CD4+ T-cells (PBS n = 4; DRQ n = 5). (D) Frequency of FOXP3+ T cells out of total CD4+ T cells (PBS n = 4; DRQ n = 5). (E) Frequency of CD8+ T-cells (PBS n = 4; DRQ n = 5). ns p > 0.05, * p < 0.05, ** p < 0.005. (TIF) [file pbio.3002905.s003.tif]

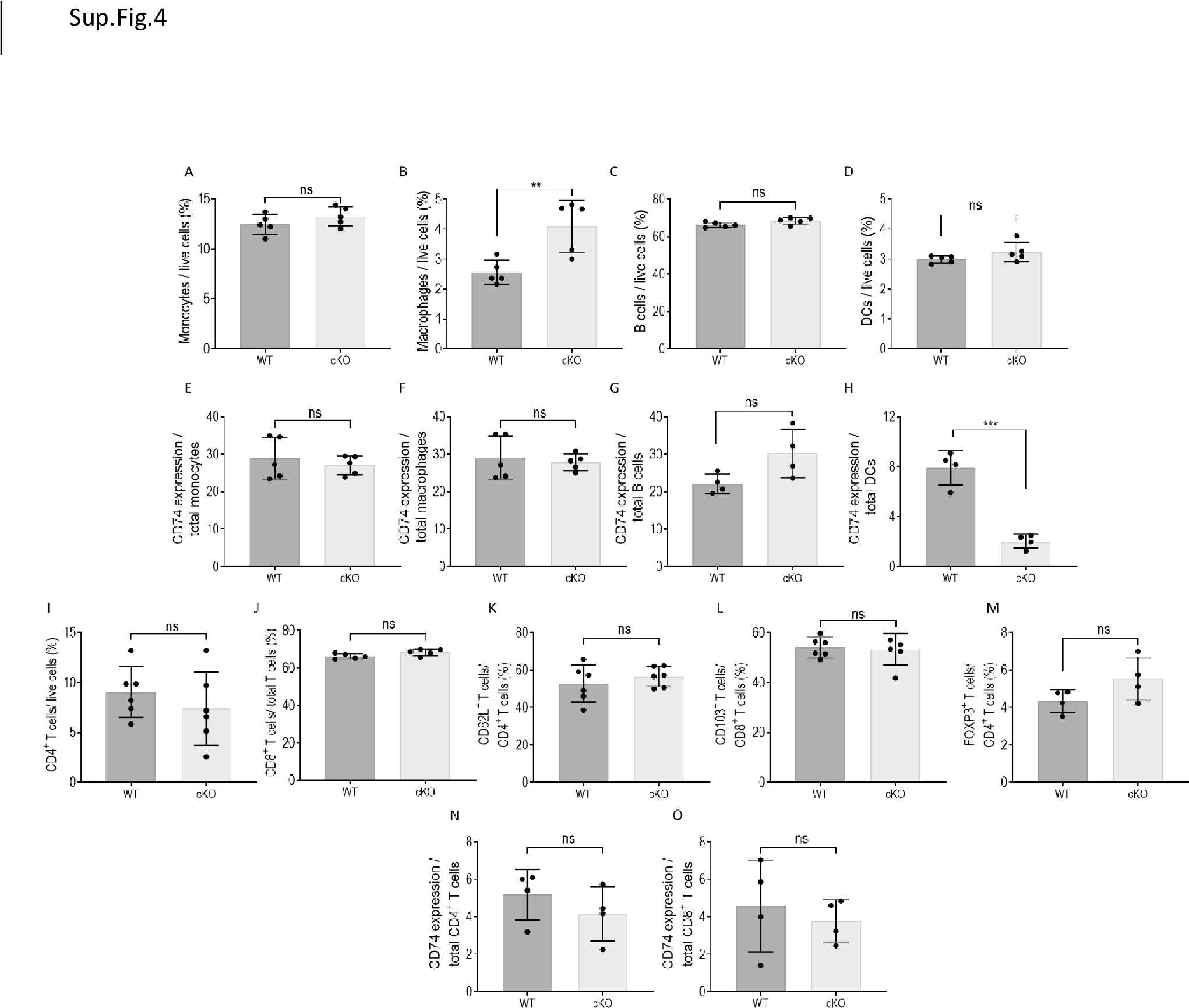

Supplement: S4 Fig — CD74 deficiency in DC specifically affects the dendritic cells population. (A-O) Female 6-week- old CD11c-Cre x CD74flox x CD74 flox mice were killed, spleens were harvested, processed to a single cell suspension, and total PBMCs were isolated. (A) Mean and SD percentage of monocytes out of total live cells (WT n = 5, cKO n = 5). (B) Mean and SD percentage of macrophages out of total live cells (WT n = 5, cKO n = 5). (C) Mean and SD percentage of B cells out of total live cells (WT n = 5, cKO n = 5). (D) Mean and SD percentage of dendritic cells out of total live cells (WT n = 5; CKO n = 5). (E–H) Mean and SD percentage of CD74 expression on monocytes (E), macrophages (F), B cells (G), and DCs (H) (WT n = 6; cKO n = 6). (I) Mean and SD percentage of CD4+ T cells out of total live cells (WT n = 6; cKO n = 6). (J) Mean and SD percentage of CD8+ T cells out of total live cells (WT n = 6; cKO n = 6). (K) Mean and SD percentage of CD62L+ T cells out of total CD4+ T cells (WT n = 6; cKO n = 6). (L) Mean and SD percentage of CD103+ T cells out of total CD8+ T cells (WT n = 6; cKO n = 6). (M) Mean and SD percentage of FOXP3+ T cells out of total CD4+ T cells (WT n = 4; cKO n = 4). (N, 0) Expression of CD74 in the CD4+ (N) and CD8+ (O) populations. ns p > 0.05, **P < 0.005, ***P < 0.0005. (TIF) [file pbio.3002905.s004.tif]

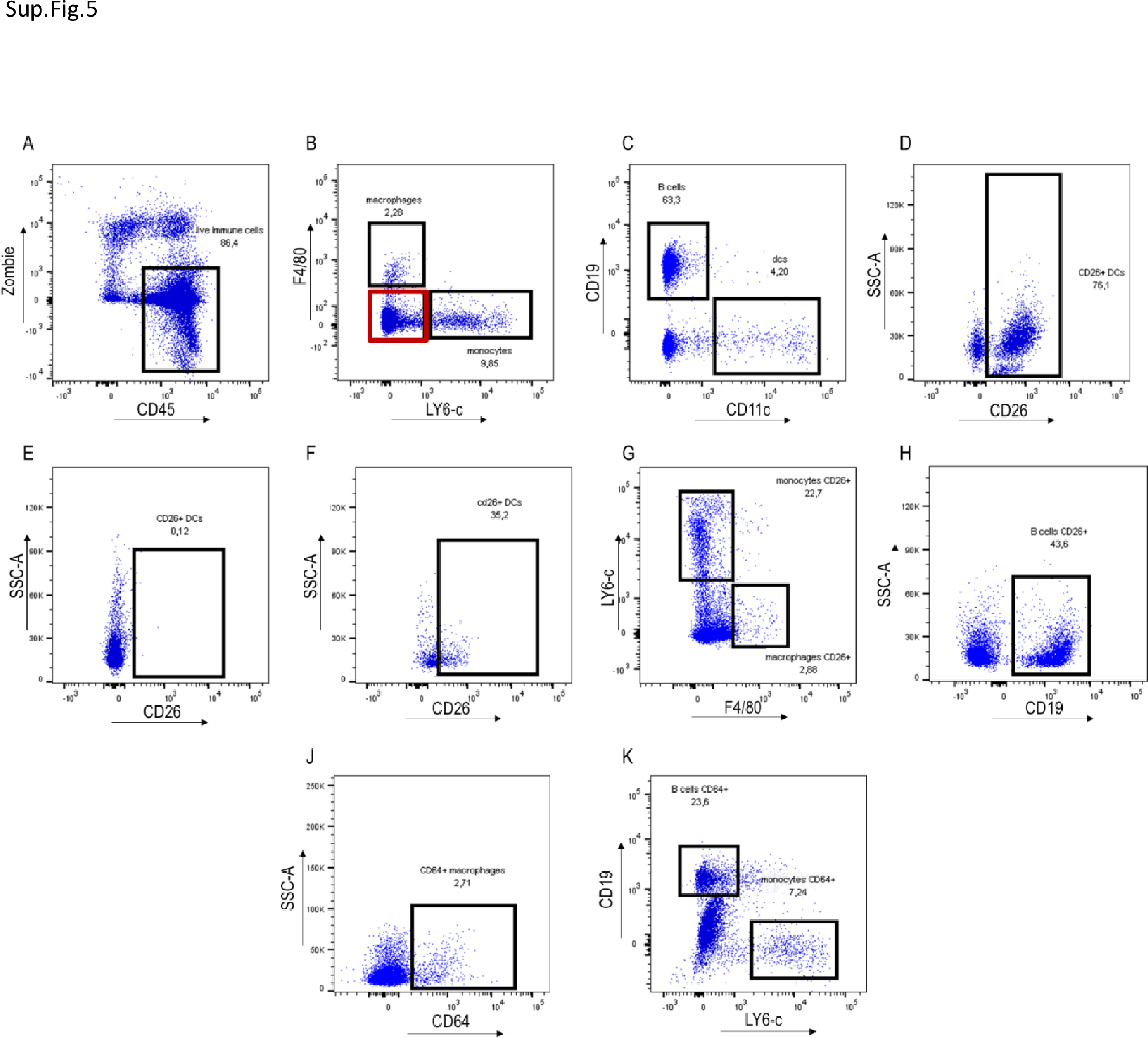

Supplement: S5 Fig — (A–K) PBMCs from the spleen of naïve mice were analyzed by flow cytometry. (A) Dead cells were excluded from analysis by Zombie Live/Dead staining. (B) Macrophages and monocytes were gated for F4/80 and LY-6c, respectively. (C) The double negative population was analyzed for CD19 and CD11c to detect DC and B cells. (D) DCs obtained in panel C were analyzed for CD26. (E, F) The CD45+ population was gated for CD26 as a dendritic cell marker. (G, H) CD26+ DCs were analyzed for F4/80, LY-6c, and CD19. (J) The CD45+ population was gated for CD64 as a macrophage marker. (K) CD64+ macrophages were analyzed for CD19 and LY-6c expression. The FCS files uploaded to FlowRepository (http://flowrepository.org/id/FR-FCM-Z8ES). (TIF) [file pbio.3002905.s005.tif]

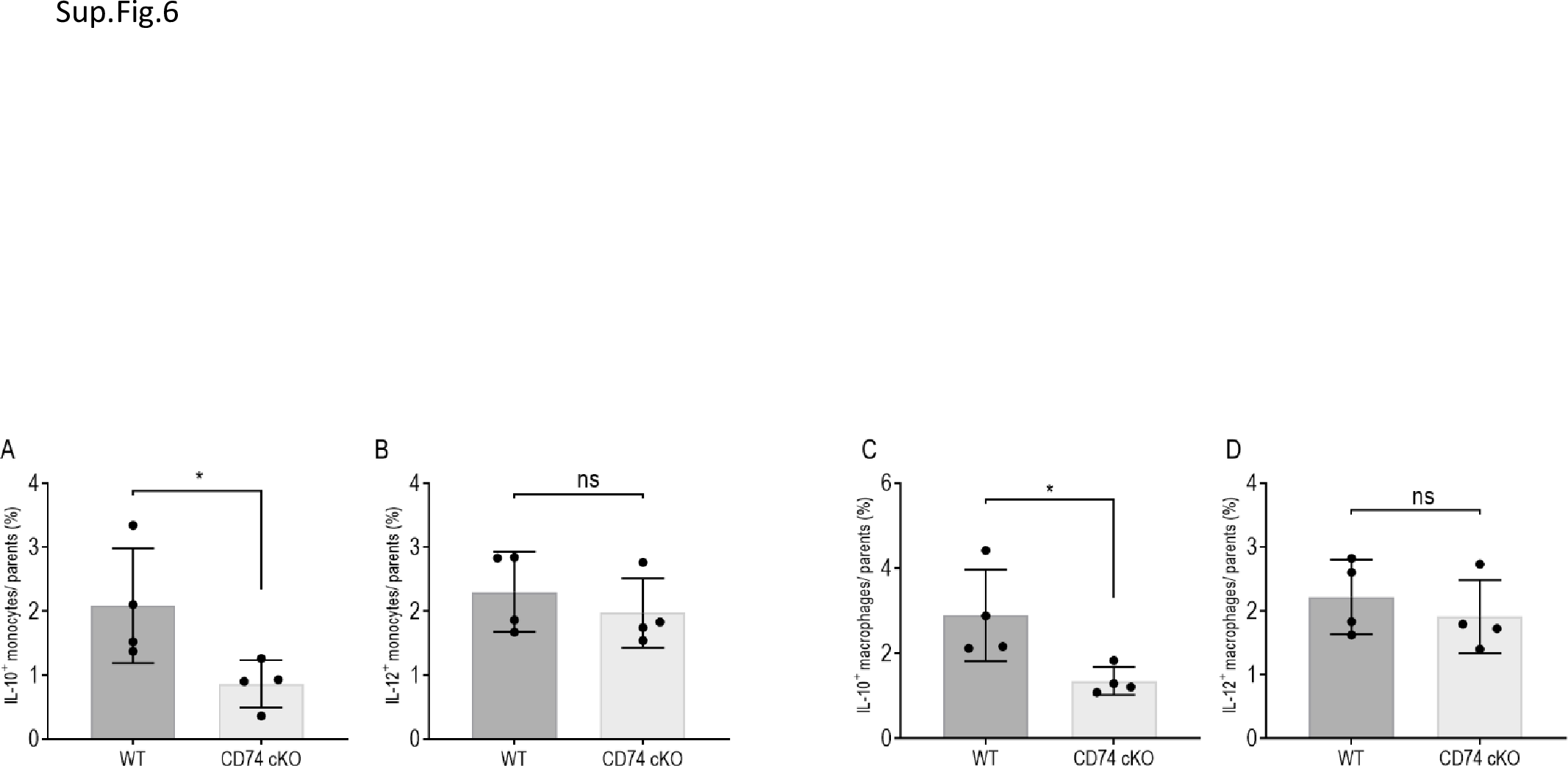

Supplement: S6 Fig — Conditional KO of CD74 in DCs impacts the IL-10 release from monocytes and macrophages. (A–D) Female 6-week-old CD11c-Cre x CD74flox x CD74 flox mice were injected with 5 * 105 E0771 cells into each of the fourth mammary pads. After 21 days, mice were killed, and tumors were harvested, processed to a single cell suspension, and total PBMCs from the tumor site were isolated. Cells were then activated with PIM and analyzed by flow cytometry. (A) Mean and SD percentage of IL-10+ monocytes out of total monocytes (WT n = 4, cKO n = 4). (B) Mean and SD percentage of IL-12+ monocytes out of the total population (WT n = 4, cKO n = 4). (C) Mean and SD percentage of IL-10+ macrophages out of total macrophages (WT n = 4, cKO n = 4). (D) Mean and SD percentage of IL-12+ macrophages out of total (WT n = 4, cKO n = 4). ns >0.05, * P < 0.05. (TIF) [file pbio.3002905.s006.tif]

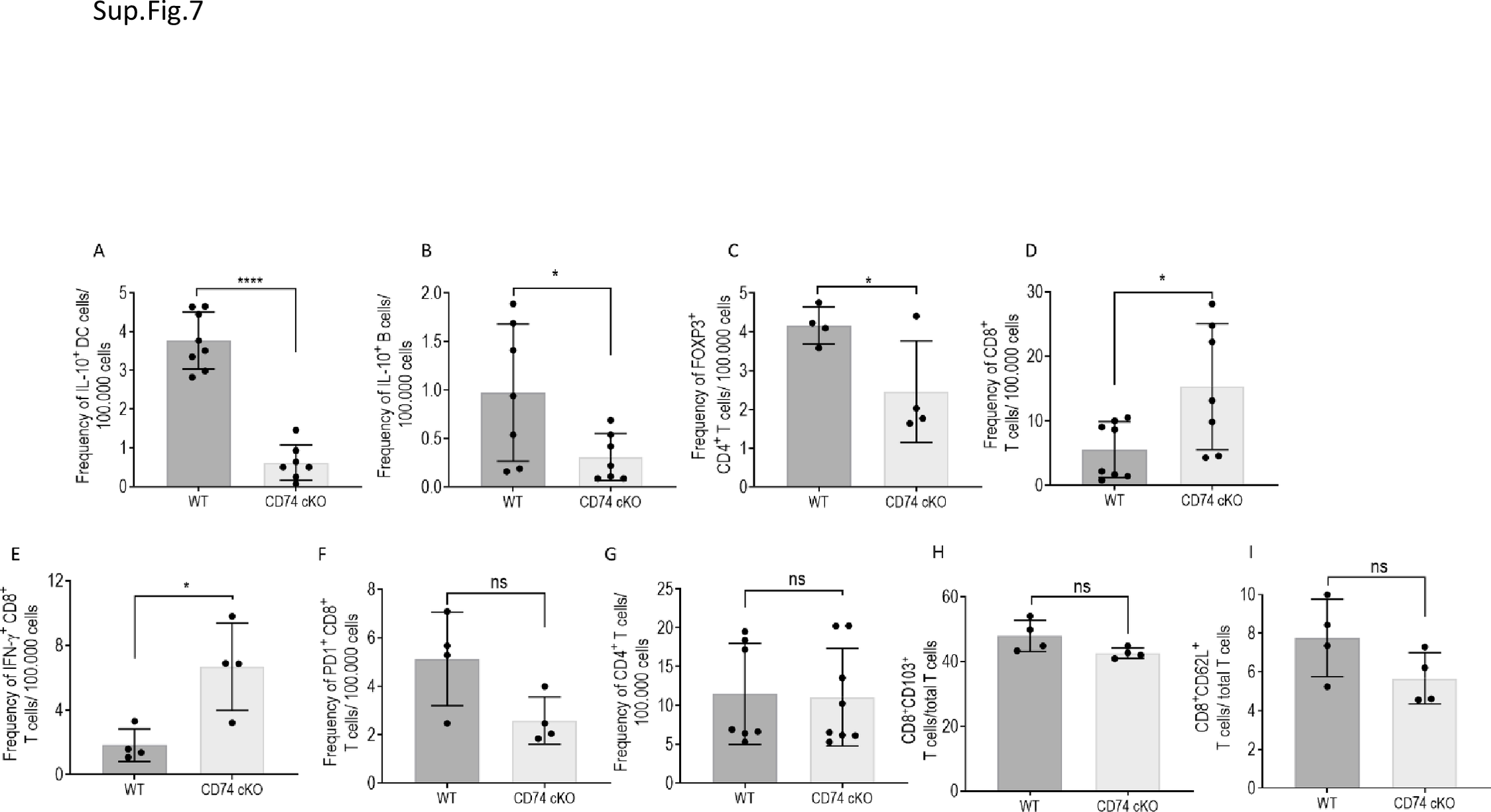

Supplement: S7 Fig — CD74 conditional KO in dendritic cells reduces the frequency of tumor-infiltrating immunosuppressive cells. (A–I) Female 6-week-old CD11c-Cre x CD74flox x CD74 flox mice were injected with 5 * 105 E0771 cells into each of the fourth mammary pads. After 21 days, mice were killed, and tumors were harvested, processed to a single-cell suspension, and total PBMCs from the tumor site were isolated. Cells were then activated with PIM and analyzed by flow cytometry. (A) Frequency of IL-10+ DCs in the tumor site (WT n = 8; CD74 cKO n = 7). (B) Frequency of IL-10+ B cells out of total B cells (WT n = 7; cKO n = 7). (C) Frequency of FOXP3+ T cells out of total CD4+ T cells (WT n = 4; CD74 cKO n = 4). (D) Frequency of CD 8+ T-cells (WT n = 8; CD74 cKO n = 7). (E) Frequency of IFN-γ+ T cells out of total CD8+ T cells (WT n = 4; CD74 cKO n = 4). (F) Frequency of PD1+ T cells out of total CD8+ T cells (WT n = 4; CD74 cKO n = 4). (G) Frequency of CD 4+ T-cells (WT n = 7; CD74 cKO n = 8). (H) Frequency of CD103+ T cells out of total CD8+ T cells (WT n = 4; CD74 cKO n = 4). (I) Frequency of CD62L+ T cells out of total CD8+ T cells (WT n = 4; CD74 cKO n = 4). ns p > 0.05, *p < 0.05, ****p < 0.00005. (TIF) [file pbio.3002905.s007.tif]

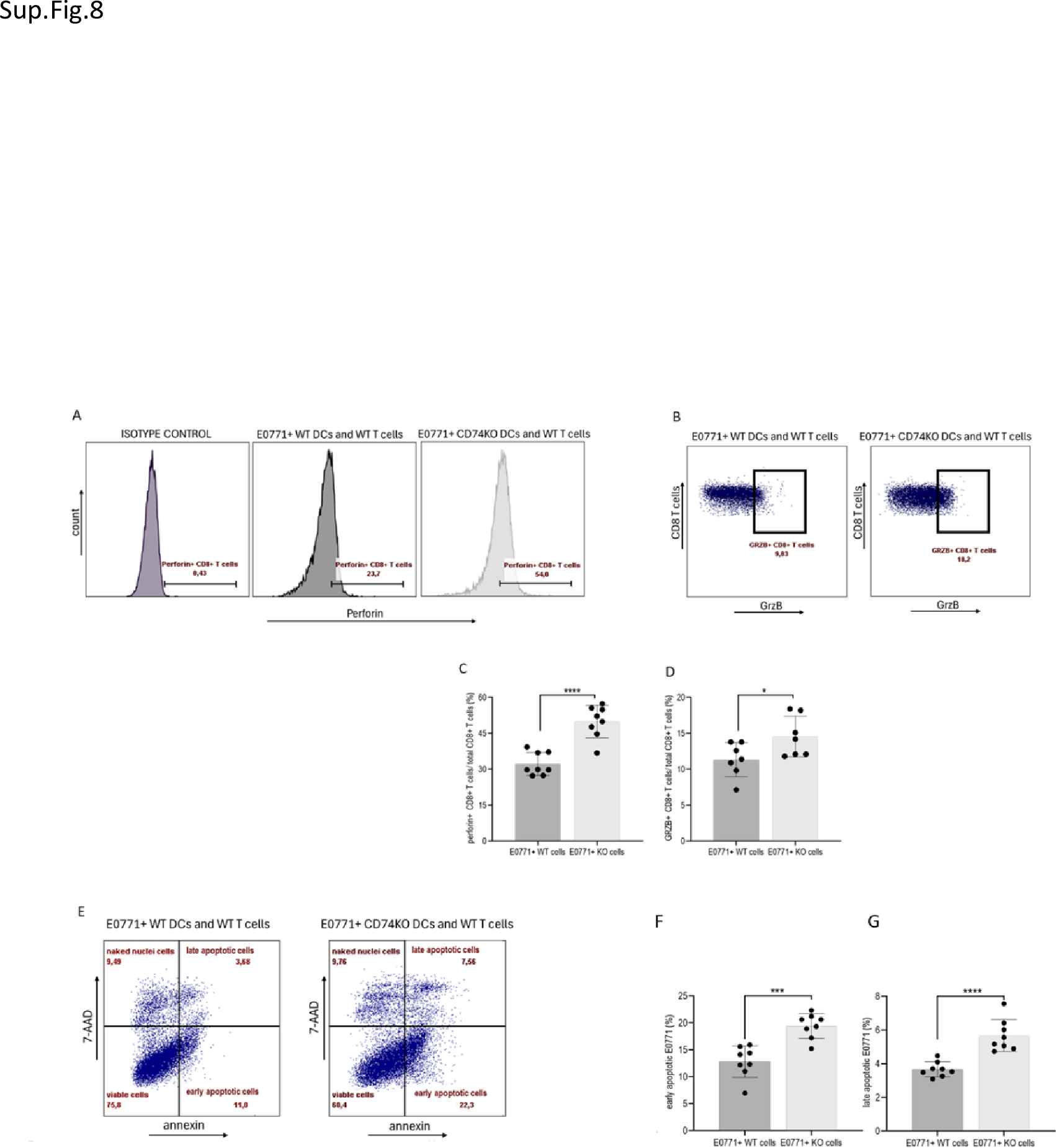

Supplement: S8 Fig — CD74 inhibition enhances the T cells killing activity. (A–G) Naïve T cells were cultured in the presence of either WT or CD74 -/- DCs and E0771 for 48H. The ability of CD8+ cells to release perforin and GrzB was evaluated by FACS. (A–D) CD74 -/- DCs better educated the T cells to release perforin compared to the WT DCs (A, C), and GrzB (B, D). (E–G) Furthermore, T cells previously educated by CD74 -/- DCs, induced an increased killing of the E0771 cells, with major amount of both early and late apoptotic E0771 (F, G) * p < 0.05,, ***p < 0.0005, ****p < 0.00005. The FCS files uploaded to FlowRepository (http://flowrepository.org/id/FR-FCM-Z8ES). (TIF) [file pbio.3002905.s008.tif]

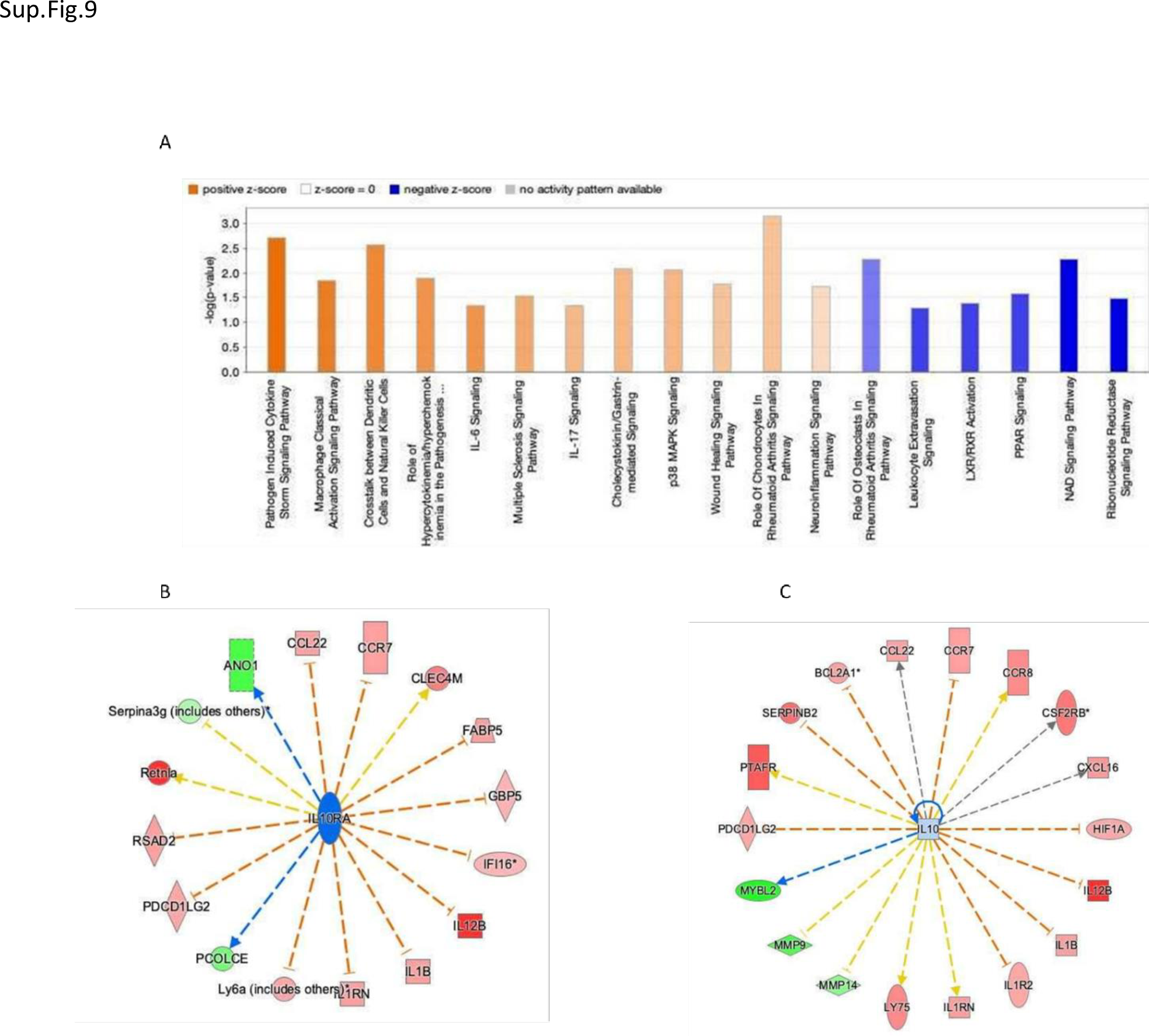

Supplement: S9 Fig — (A–C) RNA- seq analysis. Female 6 weeks old C57BL/6 mice were injected with 5 * 105 E0771 cells into each of the fourth mammary pads (total of 2 mammary pads per mouse). After 10 days, DRQ was injected intravenously for 4 consecutive days (days 10–13). After 21 days, tumor sizes were measured and mice killed. Tumors were processed into single-cell suspension and DCs were sorted from the tumor microenvironment of mice treated either with PBS or DRQ. Four replicates were used from each group. (A) Visualization of the Ingenuity Pathway Analysis (IPA) where the relevant pathways are shown ordered by significance (p value), calculated in IPA by right-tailed Fischer’s exact t test. The pro-inflammatory pathways show a positive z-score indicating that pathway activity is increased in DRQ versus PBS-treated mice. (B) IPA Upstream Regulator Analysis was used to predict the upstream regulators responsible for the gene expression changes observed. IL-10 receptor is shown to be down-regulated in DCs treated with DRQ. (C) Image depicts the gene interactions, where genes shown in red are up-regulated, while genes shown in green are suppressed. Color intensity is relative to gene expression. Genes related to immunogenic response of DCs are increased in DRQ versus PBS treated mice. (TIF) [file pbio.3002905.s009.tif]
