# Supplementary material for: CD74 promotes the formation of an immunosuppressive tumor microenvironment in triple-negative breast cancer in mice by inducing the expansion of tolerogenic dendritic cells and regulatory B cells
Source: PLoS Biol. 2024 Nov 22;22(11):e3002905. doi: 10.1371/journal.pbio.3002905 (PMC11623796; doi:10.1371/journal.pbio.3002905)
Supplement: S1 Table — (DOCX) [file pbio.3002905.s010.docx]

Sup.Table1

| **Antibody** | **Fluorophore** | **Company** | | |
| --- | --- | --- | --- | --- |
| CD19 | PE-cy7 | Invitrogen | | |
|  | BV711 | Biolegend | | |
| B220 | FITC | Biolegend | | |
|  | PB | Biolegend | | |
| F4/80 | PB | Miltenyi | | |
|  | FITC | Biolegend |  | |
|  | BV711 | Biolegend |  |  |
| Ly6-c | BV711 | Biolegend | | |
| IL-10 | PE | Biolegend | | |
|  | BV-421 | BD | | |
| CD80 | Pe-cy7 | Biolegend | |  |
|  | FITC | Biolegend | |  |
| CD11C | PE | Biolegend | | |
| IL-12 | Pe-cy7 | Biolegend | | |
| CD4 | FITC | Biolegend | | |
| CD8 | PE-cy7 | Invitrogen | | |
| FOXP3 | PE | Thermofisher | | |
| IFN-γ | PB | Biolegend | | |
| CD74 | FITC | R&D System | | |
| ZOMBIE | APC-Cy7 | Biolegend |  | |
|  | PB | Biolegend |  |  |
| CD3 | FITC | Biolegend | | |
| IL-1β | APC | Termofisher | | |
| CD45 | PERCP | Thermofisher | | |
| Perforin | PE | Biolegend | | |
| GRZ-B | PB | Biolegend | | |
| Annexin | FITC | Biolegend | | |
| 7-AAD | PERCP | BD | | |
| CD26 | APC | Biolegend | | |
| CD64 | PE | Biolegend | | |
| CD62L | FITC | Biolegend | | |
| CD103 | PB | Biolegend | | |
| CD19 | Human-unconjugated | Abcam | | |
| CD11c | Human-unconjugated | Abcam | | |
| CD74 | Human-unconjugated | Abcam | | |
