# Supplementary material for: CD74 promotes the formation of an immunosuppressive tumor microenvironment in triple-negative breast cancer in mice by inducing the expansion of tolerogenic dendritic cells and regulatory B cells
Source: PLoS Biol. 2024 Nov 22;22(11):e3002905. doi: 10.1371/journal.pbio.3002905 (PMC11623796; doi:10.1371/journal.pbio.3002905)
Supplement: S2 Table — (DOCX) [file pbio.3002905.s011.docx]

**Table**: qRT-PCR primer list.

| Organism | Gene | Forward | Reverse |
| --- | --- | --- | --- |
| Mouse | SP1 | TTG AAA AAG GAG TTG GTG GC | TGC TGG TTC TGT AAG TTG GG |
| Mouse | IL-1β | GAAATGCCACCTTTTGACAGTG | TGGATGCTCTCATCAGGACAG |
| Mouse | IL-10 | ATTTGAATTCCCTGGGTGAGAAG | CACAGGGGAGAAATCGATGACA |
| Mouse | MIF | GTTTCTGTCGGAGCTCAC | AGCGAAGGTGGAACCGTTCCA |
| Mouse | SP1 Promoter Area | CTTGCCTCGTCAGCGTCC | GTGGACTCATCCTTACCGCTC |
| Mouse | IL-1β Promoter Area | AAGAGGCTATTGCTACCCTGA | TGTCCAACTTGTTTTCCCTCCC |
| Mouse | IL-1β Genebody | TGTGAAGTAAGCAGCAGAATTTG | ACACTTCACTTCGTCGTCGTCTTAAC |
