# Supplementary material for: CD74 promotes the formation of an immunosuppressive tumor microenvironment in triple-negative breast cancer in mice by inducing the expansion of tolerogenic dendritic cells and regulatory B cells
Source: PLoS Biol. 2024 Nov 22;22(11):e3002905. doi: 10.1371/journal.pbio.3002905 (PMC11623796; doi:10.1371/journal.pbio.3002905)
Supplement: Attachment — Submitted filename: letter for reviewers 24-7 final.docx [file pbio.3002905.s027.docx]

Response to Reviewers' comments

Rev. 1:

Overall, the manuscript was an easy read--the findings were presented clearly and in a logical order. The authors did a good job of demonstrating a novel role for CD74--as a regulator of DC and B cell expansion. Although the idea of CD74 as a potential therapeutic target for TNBC is not a new one, the mechanism--CD74 promoting the formation of an immune-suppressed TME via increased expansion of tolerogenic DCs--is new and exciting. A few minor suggestions to improve the paper:

(1) Provide more human data and expand on the findings that loss of CD74 resulted in enhanced T-cell mediated cytotoxicity. Perhaps the authors could have done some IHC/IF of human tumors demonstrating a relationship between CD74 expression and the amount of tolerogenic DCs and B cells.

An immunohistochemistry analysis of biopsies from TNBC patients vs healthy controls was added to the manuscript. As shown in the new Fig. 1A, a massive infiltration of immune cells was detected in the TME of TNBC patients compared to their levels in the healthy organ. In addition, these cells, and more specifically DCs and B cells, expressed higher levels of CD74 compared to its levels on cells from the healthy donor biopsy. These results further suggest that CD74 has a role in the malignant microenvironment.

(2) Also, in addition to the levels of IFNg, the authors could have included a T-cell killing assay to demonstrate how the loss of CD74 results in enhanced CD8+T cell activity.

We added an analysis of the perforin and GrzB release from T cells that were incubated and educated with WT or CD74-deficient DCS (new Supplementary Fig. 8 A-D) and the tumor cell-killing response (New supplementary Fig. 8 E-G). T cells activated by CD74 KO DCs released more perforin (Sup. Fig. 8 A, C), granzyme B (Sup. Fig. 8 B, D) (Fig. 6 I) induced more apoptosis of E0771 compared to the T cells activated by WT DCs (Sup. Fig. 8 E-G), resulting in reduced total viability of cancer cells (Fig. 6J). Thus, CD74 regulates the functionality of DCs in the TME.

(3) One last minor comment: Figure 6A is lacking a key to indicate what the bars represent, unless the key in figure 6B is also to be referenced for figure 6A.

We added a key to the figure.

Rev. 2:

General Comments:

Pellegrino [et.al](http://et.al/). presented a very interesting and original research paper describing the role CD74 expressed in dendritic cells on regulating the immune suppression in the tumoral microenvironment. Still, I believe there are some experiments that need additional controls to be ready for publishing.

Major Comments:

On Fig.1: It is not clear (also in Fig. 1 legend) which kind of data is the embedded subplot in 1A. Is it redundant with 1C? in this case leave 1C only.

The subplot embedded in new Fig. 2A indicates the area under the curve, which represents the total amount of the measured value over the interval. New Fig. 2C compares the tumor volume between the WT and the CD74 KO mice at the end of the experiment.

On page 5 it is claimed: "...., we next wished to determine whether the reduced tumor load detected in the CD74 deficient mice results from its function as a MIF receptor, or whether it is due to the role of CD74 in antigen presentation, controlling T cell numbers".
First, It is not clear if authors want to determine if the effect of CD74 full KO mice over tumor growth is a) a general effect of having less B and T cells "Since deficiency of CD74 results in a reduced number of mature B cells and CD4+ T cells [37]" as they describe, or b) antigen presentation is acutely involved in the anti-tumoral effect under the full CD74 KO. Authors should clarify this.

Second, to address if tumor growth depends on CD74 functioning as a MIF receptor and not as a regulator of antigen presentation, authors grow tumors in the presence of CD74 ligand inhibitor DRQ vs. control (veh). Authors show that DRQ treatment also inhibits tumor growth, as in the full KO mice, claiming that "These results suggest that MIF binding to CD74 positively regulates the tumor-suppressive ME in TNBC"..

CD74 is a cell surface receptor with dual functions. It is a chaperone for MHC II, which regulates antigen presentation, and a receptor for MIF. Full deletion of CD74 results in an accumulation of immature B cells, and a reduced number of CD4+ T cells. In the current study, we distinguish between these two roles to understand which pathway is affected by CD74 in tumor induction and progression. MHC class II molecules play a critical role in both thymic selection and peripheral expansion of CD4 + T lymphocytes. CD74-deficient mice exhibit markedly decreased levels of class II expression and a dramatic change in peptide occupancy of class II surface antigens [1]. The thymic microenvironment is important during T-cell differentiation, and CD74 contributes to class II expression by thymic stromal cells responsible for positive selection. In its absence, class II expression is affected both quantitatively and qualitatively.

To determine whether antigen presentation controls tumor development, CD74 function was blocked with DRhQ, a partial MHC class II construct that inhibits ligand binding to CD74 and downregulates CD74 function as a MIF receptor on the cell surface, and not its role in antigen presentation. To interfere with antigen presentation, DRhQ would have to bind CD74 in the ER, the site of CD74 and nascent Class II association, and be tightly bound to the chaperone once reaching the endosomal compartment [2-4]. Blocking CD74 reduced both tumor growth and tumor volume (Fig. 2 J-K). Analysis of immune cells in the TME showed that this treatment elevated the percentage of immunogenic DC, resulting in a downregulation of tolerogenic IL-10^+^ DC cells (tol-DCs; Fig 2L; Sup. Fig. 3A) and upregulation of the IL-12 expressing DC population (Fig. 2M) in the TME. Furthermore, a decrease in infiltrating Bregs and Tregs was observed in the DRQ-treated mice (Fig. 2 N-O; Sup. Fig. 3 B-D). These results suggest that blocking the CD74 MIF receptor activity and not the chaperone function of MHC II regulates tumor growth and immune cell activity.

To further determine whether the lack of CD4 T cells regulates tumor progression, we analyzed tumor development in conditional KO mice, in which CD74 is absent in CD23^+^ mature B cells and CD11c^+^ dendritic cell populations. The T cell population in these mice is normal. Analysis of tumor progression in these mice revealed that T cells did not play a role in the CD74 dependent regulation, further suggesting that the MIF binding activity of CD74 regulates tumor development and progression.

On Fig.2 authors show results regarding the dependency on CD74 of tol DC and reg B expansions in the presence of EO771 tumor cells. Authors should calculate the fold values of the -EO771 columns dividing each experimental result by the mean of each experiment to show data dispersion, as I guess they did for the +EO771 columns.
We revised these figures according to the reviewer’s suggestion.

On page 5, the authors claim " Thus, the MIF secreted from the malignant cells plays a crucial role in the regulation of tol-DC and Breg expansion". This claim comes from the observation that when co-culturing MIF deficient vs. control tumor cells, tol-DC and Breg expansion is dependent on tumoral cell MIF. To claim "secreted", authors should show that the culture of B or DC cells with tumoral cell supernatant under these conditions (MIF depleted by siRNA vs. control), also reproduce the same dependencies on MIF regarding tol-DC and Breg expansions.

The reviewer is correct, and we therefore changed the sentence to the following: “MIF expression in the malignant cells plays a crucial role in the regulation of tol-DC and Breg expansion”.

In the ChIP experiments described in Fig.7D & 8 D-F, it will be very important to know if the SP1 signal is specific for the SP1 binding site of the promoter region of IL-1β. For this, a region of DNA that you do not expect to be enriched (e.g. a control region of the IL-1β gene where SP1 do not be expected to bind) and thus do not be expected to be amplified by qPCR, should be included in the procedure (negative control). A ratio of the signal found in the SP1 binding region over the one measured on the negative control region can be used as the SP1 binding measurement between the different experimental conditions. All statements regarding CD74 pathway regulating the transcriptional function of SP1 over IL1B depends on this ChIP control to be performed.

To address the reviewer’s concern, we designed a sequence for a non-coding segment in the gene-body of IL-1β. Binding of CD74 to the promoter versus the non-coding regions was compared. SP1, acting as a transcription factor, could bind the IL-1β promotor upon MIF activation, downregulating its transcription (new Fig. 9F), and not to a non-coding region in the gene body (new Fig.9H), confirming that SP1 specifically binds the IL-1β promotor.

In general, for all the figures in which the results of in vitro protocols are displayed: the described in vitro experiments have complex schemes and sometimes is not clear from the written descriptions the exact protocol used. It will help a lot to the reader to have in every figure that uses an in vitro assay a graphical scheme describing the different steps in each experiment. Some of these protocols are described in the methods section but are general and did not define the specifics of each experiment.
We added the suggested schemes for Fig. 2 and Fig. 8.

In general, for all the plots were all control treatment measurements represented by dots are in the same Y level, equal to the mean (e.g.: 6D): why is so? Can you explain this? I think the correct way to proceed is the next one: Take the mean of all control measurements (mean control), if you divide every control measurement by the mean control, that will give you the data of relativized control measurements, which should have a dispersion. Same with the treatment measurements: divide every treatment measurement by the mean control and that will give you the data of relativized treatment measurements and now you can calculate the treatment mean. Also you can use this relativized data to calculate for statistical significance.

We changed the calculation of fold change for the control measurements, dividing each value by the average of the control, as the reviewer suggested. Nevertheless, for the results of the qRT-PCR analysis, controls were normalized to 1, in order to more clearly indicate the direction of the gene expression changes in the CD74 KO or treated cells.

Minor Comments:

On page 4 there is a typo, change pregulated to regulated.
We corrected the typo.

On page 5 it is claimed: "..., but the cytotoxic activity of CD8+T cells was induced in the TME of CD74 KO mice". It is not clear which Figure depicts this, please clarify.

This statement refers to Sup. Fig. 2G, as is now clarified in the text.


Rev. 3:

MIF is an inflammatory cytokine and atypical chemokine that signals through CD74 and through the chemokine receptors CXCR2 and 4 to promote inflammatory and tumorigenic processes. Here, Pellegrino and colleagues have studied the specific role of the MIF/CD74 axis in triple negative breast cancer (TNBC) and in the tumor microenvironment. They demonstrate that CD74 expressed on CD11c+ cells (i.e. dendritic cells), regulates tumor growth through the control of the cross-talk between the tumor-infiltrating tolerant (tol)-DCs and regulatory B cells (Bregs). The study is comprehensive, well-controlled and the manuscript well written and of general interest for biologists and biomedical researchers. The findings suggest the MIF/CD74 axis as a novel therapeutic target in TNBC.

Main comments

Upon MIF binding, CD74 forms a cell surface complex with CD44, which is essential for the MIF-induced signaling cascade. The signaling pathway involves Syk tyrosine kinase and PI3K/Akt activation, which leads to CD74 intramembrane cleavage and the release of the CD74 intracellular domain (CD74-ICD). -Regarding a statement in the introduction and - generally - conceptually, it would be interesting for the reader to know whether the MIF-triggered CD74-ICD mechanism has actually been shown to involve CD44. In other words, is ICD generation blocked in Cd44-deficient cells? If this hasn't been shown, I would suggest to down-tone the phrase.
Referencing needs to be improved and updated; some key papers from the MIF and CD74 field are missing. For example, a relatively recent paper reporting on CD74 in DCs is missing in the reference list. This paper should be included and accordingly discussed.

We have previously shown that in B cells, MIF stimulation elevated the degree of intramembrane cleavage and liberation of the CD74 cytosolic domain. The specific augmentation of CD74-ICD release was abolished in CD44-deficient cells [5]. Based on these findings, we indicated that CD44 is essential for the MIF-induced cascade.

In addition, the bibliography was updated.

Figure 1L ff.: based on the pharmacological blocking experiment with DRQ, authors suggest that "MIF binding to CD74 positively regulates the tumor-suppressive ME in TNBC". Can they please discuss (or explain in the rebuttal letter), as to how DRQ might also block MIF-2 responses, and how/why it doesn't interfere with the MHC II chaperone activity of CD74.
DRhQ is a partial MHC class II construct that inhibits ligand binding to CD74 and downregulates CD74 function as a MIF receptor on the cell surface, and does not affect its role in antigen presentation. To interfere with antigen presentation, DRhQ would have to bind CD74 in the ER, the site of association between CD74 and nascent Class II, and remain tightly bound to the chaperone once reaching the endosomal compartment [2-4]. We added this explanation to the Discussion section.

DRhQ was shown to inhibit the function of both MIF-1 and MIF-2 [6], suggesting a potential role for MIF-2 in the tumor microenvironment. However, in the current study, we did not analyze the effect of MIF-2 on the immunosuppressive ME. Such a study will be conducted in the future.

Figure 2: the co-culture experiment in Figure 2 would highly benefit from an experimental flow chart ("cartoon") outlining the co-culture set up. I would suggest to include this scheme as new Flow charts were added to the new Fig. 2 and to the new Fig. 8.

Figure 2 ff.: the MIF siRNA approach in Figure 2F is an elegant approach to show that tumor cell secreted MIF is involved. Still as MIF knockdown may more generally alter tumor cell behavior, consider using (or at least discussing) the usual approaches to interfere with secreted MIF, i.e. using a neutralizing MIF Ab or MIF SMDs such as 4-IPP. Plus: were MIF levels in the sups actually controlled, e.g. by ELISA?

Cancerous cells produce and release high levels of MIF. These cells are incubated with media and sera that also contain high levels of MIF. Therefore, to directly show that MIF derived from the malignant cells, and not that present in the media, regulates immunosuppressive cell expansion, we chose to specifically downregulate MIF expression in the cancer cells. Using a neutralizing MIF antibody or a MIF antagonist would block MIF derived from both the media and the cells. Thus, reducing the MIF levels directly in the E0771 cells provided a more accurate indication regarding the source of MIF.

Figure 6: I understand that normalization was necessary in these experiments. Still, consider representing the error bars for the PBS controls.
We revised the figures, and normalized the control to the average to show the dispersion.

Discussion: Cd74-deficiency leads to decreased numbers of Bregs, Tregs, and IL-10+ macrophages in the TME, overall suggesting that CD74 (on DCs) promotes an anti-inflammatory profile in the TME. Authors may want to discuss how this compares to the otherwise and generally known role of CD74 in promoting inflammation. In other words, it would be good to even better discuss the mechanisms that specifically lead to this kind of inverse phenotype in the specific setting of the TME.

Although CD74 was shown to be associated with a pro-inflammatory response in several diseases, including pancreatic cancer, its effect is context-dependent, indicating that its role can change according to specific environmental cues. Indeed, it was demonstrated that CD74 regulates an anti-inflammatory pathway in melanoma that confers a tolerogenic phenotype on both DCs and macrophages; supports regulatory T-cell accumulation in several tumors; and hampers the M1 polarization of microglia in brain metastasis from NSCLC. Therefore, we would not consider this effect as a reverse phenotype, but a phenotype specifically induced in TNBC [7-9].

Minor points

1. Page 4, typo: CD74 expression is pregulated in various....

We corrected the typo.

2. Consider citing Verjans et al, BMC Cancer 2009; which shows specifically an involvement of MIF in BC.

We added the reference.

3. Figure 8: MIF or MIT?

The new Fig. 9 E,F (previous figure 8) demonstrates the Chip qRT-PCR, in which the binding of CD74 to SP1 promotor with or without MIF treatment was compared (Fig. 9E), and the binding of SP1 to the IL-1β promotor was analyzed following a treatment with either DRQ or MIF (the new Fig.9 F). The new Fig. 9 A,B,C,G represent instead the comparison between DMSO and the SP1 inhibitor, MIT.

References

1.	Kenty G, Martin WD, Van Kaer L, Bikoff EK. MHC class II expression in double mutant mice lacking invariant chain and DM functions. J Immunol. 1998;160:606-14.

2.	Meza-Romero R, Benedek G, Gerstner G, Kent G, Nguyen H, Offner H, et al. Increased CD74 binding and EAE treatment efficacy of a modified DRalpha1 molecular construct. Metab Brain Dis. 2019;34(1):153-64. Epub 2018/10/26. doi: 10.1007/s11011-018-0331-2. PubMed PMID: 30353480; PubMed Central PMCID: PMCPMC6364671.

3.	Meza-Romero R, Benedek G, Leng L, Bucala R, Vandenbark AA. Predicted structure of MIF/CD74 and RTL1000/CD74 complexes. Metab Brain Dis. 2016;31(2):249-55. Epub 20160206. doi: 10.1007/s11011-016-9798-x. PubMed PMID: 26851955; PubMed Central PMCID: PMCPMC5248574.

4.	Meza-Romero R, Benedek G, Yu X, Mooney JL, Dahan R, Duvshani N, et al. HLA-DRalpha1 constructs block CD74 expression and MIF effects in experimental autoimmune encephalomyelitis. J Immunol. 2014;192(9):4164-73. doi: 10.4049/jimmunol.1303118. PubMed PMID: 24683185; PubMed Central PMCID: PMCPMC4028955.

5.	Gore Y, Starlets D, Maharshak N, Becker-Herman S, Kaneyuki U, Leng L, et al. Macrophage migration inhibitory factor (MIF) induces B cell survival by activation of a CD74/CD44 receptor complex. J Biol Chem. 2008;283:2784-92. PubMed PMID: 18056708.

6.	Matejuk A, Benedek G, Bucala R, Matejuk S, Offner H, Vandenbark AA. MIF contribution to progressive brain diseases. J Neuroinflammation. 2024;21(1):8. Epub 20240104. doi: 10.1186/s12974-023-02993-6. PubMed PMID: 38178143; PubMed Central PMCID: PMCPMC10765708.

7.	Figueiredo CR, Azevedo RA, Mousdell S, Resende-Lara PT, Ireland L, Santos A, et al. Blockade of MIF-CD74 Signalling on Macrophages and Dendritic Cells Restores the Antitumour Immune Response Against Metastatic Melanoma. Front Immunol. 2018;9:1132. Epub 20180523. doi: 10.3389/fimmu.2018.01132. PubMed PMID: 29875777; PubMed Central PMCID: PMCPMC5974174.

8.	Bonnin E, Rodrigo Riestra M, Marziali F, Mena Osuna R, Denizeau J, Maurin M, et al. CD74 supports accumulation and function of regulatory T cells in tumors. Nat Commun. 2024;15(1):3749. Epub 20240503. doi: 10.1038/s41467-024-47981-3. PubMed PMID: 38702311; PubMed Central PMCID: PMCPMC11068745.

9.	Liu L, Wang J, Wang Y, Chen L, Peng L, Bin Y, et al. Blocking the MIF-CD74 axis augments radiotherapy efficacy for brain metastasis in NSCLC via synergistically promoting microglia M1 polarization. J Exp Clin Cancer Res. 2024;43(1):128. Epub 20240429. doi: 10.1186/s13046-024-03024-9. PubMed PMID: 38685050; PubMed Central PMCID: PMCPMC11059744.
